# Criteria, Procedures, and Future Prospects of Involuntary Treatment in Psychiatry Around the World: A Narrative Review

**DOI:** 10.3389/fpsyt.2019.00271

**Published:** 2019-04-29

**Authors:** Anna Saya, Chiara Brugnoli, Gioia Piazzi, Daniela Liberato, Gregorio Di Ciaccia, Cinzia Niolu, Alberto Siracusano

**Affiliations:** ^1^Chair of Psychiatry, Department of Systems Medicine, University of Rome Tor Vergata, Rome, Italy; ^2^Psychiatry and Clinical Psychology Unit, Department of Neurosciences, Fondazione Policlinico Tor Vergata, Rome, Italy

**Keywords:** involuntary treatment, coercion, self-determination, suicide prevention, advanced directives, decision-making capacity, stigma, therapeutic relationship

## Introduction

The use of involuntary treatment in psychiatry comes with some benefits and many disadvantages for the patient’s experience and the therapeutic outcome. This review proposes to compare the procedures and criteria for involuntary psychiatric treatment around the world. We highlight the gap between legislation and practice, the patient’s experience of coercion, the repercussions on the therapeutic relationship and adherence to treatment following coercion, the role it plays in the prevention of suicide and of hetero-aggressive behavior, ethical problems, and possible alternatives to reduce the use of coercive measures.

### History and Legislation

Mental health legislation has changed significantly, starting in Europe and North America, and eventually beginning to globalize from the 1960s onward, with macroscopic exceptions. The focus shifted from explicitly expelling the mentally ill for the protection of society to curing mental illness itself. In the 19th and part of the 20th centuries, mental health laws were forged from the models for criminal procedures. Mental illness was treated as a transgression and hospitalizations resembled prison stays, under worse conditions, considering that the duration of detention for the mentally ill was undetermined (1). The world’s most famous asylum, London’s Bethlem Royal Hospital, also known as Bedlam, was established in 1307 as a general hospital and converted into an asylum for the mentally ill in 1403. Centuries later, the USA began to build asylums that also followed the idea of indefinite confinement and used methods that included seclusion, sedation, and experimental treatments with opium, without any actual benefit (1). They were custodial institutions rather than places for treatment and recovery (2). The de-institutionalization of the mentally ill in the USA began in 1960, and in 1963, President Kennedy signed an Act^1^ to facilitate the transition from asylums to community mental health centers. This contributed to a decrease in the number of hospitalized patients from 550,000 in 1950 to 30,000 in 1990 (1).

Italy also followed a custodial model of treatment, in which the mentally ill were considered exclusively with regard to the risk they posed to themselves and others, allowing for their commitment to institutions by a judicial authority (3). This codified an equivalence between criminality and mental illness. This system remained in place until the implementation of the Basaglia Law on 13 May 1978, which regulated voluntary and mandatory health checks and treatments for the mentally ill and ordered the closure of asylums (4). There was a fundamental transformation of the custodial style following the implementation of this law, which set time limits on hospitalizations or at least foresaw a future discharge.

Long-term hospitalizations seemed to cause symptoms associated with chronic mental illnesses, constituting an “institutionalization syndrome” (5). Since the 1960s, this syndrome has been noted as a psychopathological condition due to the pathogenetic effect of situations as long-term residence in closed institutions such as psychiatric hospitals and prisons, where they assumed a purely institutional role. The symptomatology includes withdrawal into oneself, apathy, regression to infantile behavior, stereotypes, and ideological slowdown. Goffman speaks about the “mortification of self” and argued that patients whose freedom was restricted suffered from the stigma of being a psychiatric patient (6).

Two separate factors inherent to modern psychiatric care concur with the change in focus from custodial to curative goals. Psychoanalysis, with the discovery of the unconscious, led to increased comprehension and integration of psychopathological phenomena, underlining the continuum between normalcy and pathology, as indicated by Freud, and reducing the strong connotation of the split element that mental illness had previously represented. The other factor is represented by psychopharmacology, specifically the improved ability to control some symptomatological manifestations and the consequent behavioral correlations.

Parallel to the transformation of psychiatry, social changes determined a radical overturning of the role of the judicial authority. Originally represented as a depository of power over the custody of mentally ill patients, the judicial authority later became a guarantor of their rights, hearing their appeals against involuntary treatment. In fact, the Council of Europe’s “White Paper on the Protection of Human Rights and Dignity of People Suffering from Mental Disorders, Especially Those Placed as Involuntary Patients in a Psychiatric Establishment” provided *inter alia* that the patient should be examined by a doctor or experienced psychiatrist, and that the admission decision should be confirmed by an independent authority. It also provided that treatment must be based on an individualized plan, discussed with the patient, and periodically reviewed by adequately qualified staff (7).

Current national laws on mental health are inspired by two concepts: the principle of *parens patriae*, which gives government the responsibility to intervene for citizens who are unable to protect their interests, and police power, which protects the safety of its citizens. Government enacts statutes for the welfare of its society, and involuntary hospitalization is placed in the broad and detailed context of how much the State can and should intervene, even to the cost of restricting the freedom of some individuals (1).

The debate regarding nonconsensual treatment centers on the issues of freedom and self-determination. In 1979, Gostin affirmed that one cannot presume that the status of a person who has been hospitalized against their will coincides with a complete loss of self-determination (8). The clinical reality suggests that residual autonomy and decisional freedom exist, even for involuntarily hospitalized patients. Along that line, Grisso and Appelbaum proposed a multidimensional approach that foresaw an assessment of the patient’s ability to consent (in other words, on their residual decision-making ability in line with the new Code of Medical Deontology as well as the Basaglia Law). The United Nations Convention on the Rights of Persons with Disabilities also highlights the importance of assessing the patient’s ability to take a reasoned decision (9).

Health care professionals and the law share a common goal: to consider the patient, as much as possible, as a partner in the decision-making process, apart from their areas of deficiency (10). Various international documents on human rights represent points of reference for State legislations. Some examples are the Principles for the Protection of Persons with Mental Illness (or MI Principles, 1991) (11), the European Convention for the Protection of Human Rights and Fundamental Freedoms (1950) (12), the Declaration of Hawaii (1983) (13), and the Ten Basic Principles for Mental Health Law published by the World Health Organization (WHO) (14, 15).

Current western legislation strongly emphasizes the protection of rights for patients with mental illness, and compulsory treatment is considered as an absolutely exceptional measure (16). Coercive treatment is an exception with respect to all other medical treatments, which, though they may be necessary, cannot disregard the requirement for informed consent and can always be refused. Danish law^2^ highlights the exceptionalism of nonconsensual treatment, allowing it only after attempts to obtain consent, coherent with the view of “minimal intrusive remedy” (17). The Lanterman–Petris–Short Act, introduced in the USA in 1967 and implemented in 1969, represented the prototype for mental health laws in many other western countries (18). This act aimed to abolish permanent admissions, improve public health, and guarantee the rights of patients with mental illness. After its implementation, the number of involuntary treatments in California remained the same, but the number of voluntary treatments, which perhaps were the result of a more frequent use of informal coercion, doubled. The law also reduced the duration of mandatory hospitalizations (2). Similarly, even though Poland had fewer involuntary treatments in the years 1996–2005, there was an increase in the absolute number of treatments due to the general increase of requests for psychiatric admissions (19). This was due, in part, to higher levels of confidence in psychiatric care.

In 1977, the World Psychiatric Association developed a code of ethics for clinical practice, named the “Declaration of Hawaii” (13). The WHO recommends that mental health treatments should be as efficient as possible; hospitalization durations should be limited to the risk posed and used only if it is the only way for the patient to receive treatment (14). The European Convention on Human Rights (ECHR) provides that forced hospitalization should remain in the guaranteed context of Article 3 “which prohibits any inhuman or degrading treatment” (20). The jurisprudence of the European Court on Human Rights (ECtHR), which is binding on the 47 Member States party to the Convention, clarified the conditions under which involuntary admissions can occur in accordance with the abovementioned Article 3, as well as Article 5, paragraphs 4 and 8 ECHR (21). These conditions require the establishment of a mental illness based on a medical evaluation, exceptionality and urgency, nature and gravity of the psychic disorder such as to justify the deprivation of liberty, measure proportional to the need for the safety of the patient and the community, and temporary limitation of the measure to the period of persistence of the illness (22).

While there are many studies regarding involuntary treatment in Europe, North America, and Oceania, there is some difficulty in finding valid recent studies for Asia, Africa, and Latin America. This imbalance can be attributed to lack of investment in the health systems where limited resources are dedicated to treatments rather than research, the disruptions of political instability and war, and public health emergencies and epidemics that direct resources away from psychiatric care.

## Methods

The articles have been chosen, both as regards historical citations and current procedures, by searching on accredited sites (such as PsychoINFO, PubMed, Google Scholar, Research Net, MedLine, and HUDOC), governmental internet sites, and databases of organizations (such as WHO MinDBank). The most recent search was conducted on 16 March 2019. Out of a total of 302 publications consulted, we selected 85 articles for our bibliography, researching, in addition to scientific articles, the legislative sources specific to each mentioned country. We eliminated articles that included topics such as specific treatments or mental illnesses. The keywords we used include involuntary treatment, involuntary admission, mental health law, and emergency admission. For the historical part, we have used legislation and articles from the second half of 1900 to the most recent available; regarding the results, the articles were selected from publications from 2000 to 2019. An up-to-date source was not available for all countries, particularly African, Middle Eastern, East Asian, and South American countries. We used mainly English language articles with high bibliographic sources that were published in international literature. Whenever possible, we included the most recent bibliographies.

## Results

It is important to study the various criteria required for involuntary treatment, and their weight, as well as the diverse procedures provided around the world. When mandatory medical treatment and hospitalization are necessary, such legislation interrupts the therapeutic relationship, changing the quality of the communication according to whether it comes from the patient’s doctor, an alternate doctor, or a judicial authority. The intervention of the latter, on the one hand, can be experienced as a persecutory intrusion in the care relationship but, on the other hand, can be seen as a guarantor of the patient’s rights (to freedom and to be heard) for its role in reviews and appeals against involuntary treatment decisions. It is useful to distinguish between situations in which the person subject to compulsory treatment is already in treatment, and when instead, it is the patient’s first contact with psychiatrists, as is frequently the case for marginalized persons. More or less restrictive criteria also influence the quantity of involuntary treatments. The direct involvement of the patient’s treating psychiatrist in the treatment (or the caregiver who may request it) has an important relational meaning. The specific weight of the danger, or need for treatment, and the assessment of the patient’s lack of decision-making capacity (carried out more frequently with regard to pharmacological treatment) affect the patient’s perception of the experience.

The various weights given to the patient’s decision-making capacity in the various national legislations are reflected in the legality of pharmacological treatments in involuntarily hospitalized patients. In some countries (such as Italy), involuntary pharmacological treatments are automatically permitted for hospitalized patients, while others require a more articulated procedure to decide on treatment options. Germany forbids an automatic association between involuntary hospitalization and involuntary pharmacological treatments, as they can be considered unconstitutional. This contrast between styles might create a kind of paradox in which patients could be admitted without consent but left without treatment.

The requirement of the presence of mental illness, with a view to balancing the principle of autonomy with that of beneficence, cannot, by itself, constitute a sufficient element for involuntary psychiatric treatment. In fact, all related regulations also mention the need for treatment, the dangerousness of the patient, or both factors. There is greater difficulty in deciding on the admission of people with mental illnesses but who are not considered dangerous because there is variability in the interpretation of the gravity of the disorder (whether it requires hospitalization) and the degree of deterioration of the patient’s decision-making capacity (18). This seems to actually lead to fewer admissions of nondangerous people for whom hospitalization is sometimes the first, albeit traumatic, moment of access to treatment. The Amsterdam Studies of Acute Psychiatry proposed a comparison of two groups of 125 patients with voluntary and mandatory treatment. Variables that distinguish between groups include social support and access to healthcare. In fact, specific cultural and socioeconomic groups more rarely covered by the mental health care system, such as migrants, more often have their first contact with mental health workers through emergency services (23).

The variety of related jurisprudence among countries relies on the type and severity of the mental disorder, which should be such that it leads to a reduction in decision-making capacity in order to justify the absence of consent.

### Europe

The UK Mental Health Act of 1983 (24) and the Welsh Mental Health Act Code of Practice (25) define “mental disorder” as “any mental disorder or mental disability” (see **Table 1**). This criterion can include mental retardation, substance abuse disorders, and personality disorders. The dangerousness criterion is sufficient for an involuntary admission in the UK (15), where there are two types of criteria based on the length of the hospitalization. Detention for assessment can last up to 28 days and requires that the patient suffer from a mental disorder, which, at the pertinent time, requires assessment for their health or safety or for the protection of others. In the second case, regarding hospitalizations for treatment up to 6 months, renewable, there are the same criteria mentioned above, with the addition of the availability of treatment. In England and Wales, procedurally, there is no immediate obligation to revise the admitting doctor’s decision. Patients may request a review to the Mental Health Review Tribunals of the decision posthospitalization. Reviews take place automatically after 6 months and then after every 3 years of continuous admission (5).

**Table 1:** Legislations, criteria, and procedures in Europe.

Country	Legislation	Diagnosis	Other criteria	Proposal	Validation	Duration	Ability to appeal
**England and Wales**	Mental Health Act 1983, amended in 2007; The Welsh Mental health code of practice	Any disorder and disability of mind	The mental disorder requires detention for assessment; detention in the interest of health and safety of patient and others; available appropriate medical treatment	Relatives or professionals; police (emergency)	Two registered medical practitioners with written recommendations (non-emergency); a mental health professional and a doctor (emergency)	Admission for assessment: 28 days; for treatment: 6 months renewable once for another 6 months	Yes; mental health review tribunal
**Northern Ireland**	Mental Health Order 1986, amended in 2018	Mental disorder as “mental illness, mental handicap or any other disorder or disability of mind”	The mental disorder requires detention for assessment; failure to detain would create serious physical harm to patient and others	The nearest parent or an approved social worker; application for assessment shall be accompanied by medical recommendation	Responsible medical officer for assessment	Assessment: 2 days; detention for medical treatment: 6 months renewable of 6 months; then further periods of 1 year	Yes; mental health review tribunal
**Scotland**	Mental health Act 2015 amended the MHA (care and treatment) 2003	Mental disorder	Medical treatment available; significant risk for the health and safety of patient and others; impaired ability to make decisions; requires treatment	Two medical practitioners; mental health officer	Tribunal hearing persons who have opportunity to make representations, of leading and producing evidence	6 months renewable for 6 months twice; subsequent further reviews: 12 months	Yes; patient’s responsible medical officer
**Italy**	Law 180 incorporated in Law 833 of 1978	Mental condition requiring urgent treatment	The person does not accept the treatments; it is not possible to take appropriate extra hospital measures	Two physicians	The mayor and authorized by the tutelary judge who is entrusted with the jurisdictional safeguard of such treatment	7 days; renewable several times at the request of the psychiatrist to the mayor	Yes; competent court
**Spain**	Ley de Enjuiciamiento Civil of 8 January 2000, Book IV, Title I. Chapter 2 art. 763	Psychological disturbance, which causes inability to take decision and care for oneself	–	Psychiatrist	Judge	–	Yes
**Portugal**	Law 36/98 and decree 35/99, which regulates the law	Person with severe psychic anomaly	Danger to themselves or others, and refuses to submit to necessary medical treatment	Legal representative; public health authorities; the Public Prosecution Service; doctors; the clinical director of an institution (if in the course of a voluntary admission)	Judge	Least restrictive possible	Yes
**Greece**	(Law 2071/1992 (hospitalization. 123/A/1992): Modernization and health system organization. Articles 94–100: involuntary hospitalization and treatment The enforcement of Law 2071/92 for Involuntary Hospitalization of Psychiatric Patients. 504: Interpretative Circular of the Supreme Court of Appeal (1996)	Diagnosis of a severe mental disorder, takes into account the patient’s need for treatment and their dangerousness	Inability to judge one’s own health interest; if the nonadmission could lead to ineffective treatment or aggravation of the disease Dangerousness criteria are sufficient	Two individual assessments by psychiatrists; the closest relative brings the report to judges authorized to police escort Public Prosecutor	Two qualified psychiatrists in 24 h then Public Prosecutor orders the patient’s admission	–	Yes
**Belgium**	Loi modifiant la loi du 26 june 1990 relayive à la protection de la personne des malades mentaux 2017	Mental disorder	Danger for the health and safety of the person and others	Any interested person	Justice of the Peace	Observation period of maximum 40 days; the duration of maintenance cannot exceed 2 years (renewable)	Yes; Justice of the peace
**The Netherlands**	Rules may provide for compulsory care to a person with a mental disorder (Obligatory mental health) 2009; Compulsory admission Act 1992	Severe mental disorders that constitute a danger to themselves or others, including severe negligence or severe social inadequacy	Imminent danger for oneself and others	Doctor; judge	Judge	3 weeks (emergency procedure) 6 months (regular procedure)	Yes
**France**	Law n ° 2013–869 (27–9–2013)	Admission at a request of a third party: mental disorder	The mental disorder makes consent impossible and requires immediate care and medical supervision	Family member, guardian, or curator of the patient	The director of the establishment on the basis of two medical certificates	After the first period of observation (72 h) a month, renewable	Yes; college of experts, departmental commission
		Admission on the decision of the representative of the state: mental disorder	People whose mental disorder require care and jeopardize the safety of people or public order	Warden Home representative	Representative of the State		
**Germany**	Different regulations and procedures for involuntary placements or treatments among Germany’s 16 federal states	Some federal states specify “psychosis”	Mental disorder and severe conditions “equivalent to psychosis” + danger (dangerous or self–destructive behavior)	“Physician” in some Federal States; “psychiatrist” or “physician experienced in psychiatry” in others	A specialist must be involved in assessment of psychiatric condition Maximum period of time between psychiatric assessment and compulsory admission different for each Federal State: ranging from 24 h to 14 days	Preliminary detention: 6 weeks regular placement 1 year, in obvious cases 2 years Reapproval of decision is 6 months (defined by Federal State of Saarland only)	Patients have the right to appeal to courts at any stage of the procedure Patients’ advocates are approved during all stages
**Switzerland**	Swiss civil code (Part three; third section; third paragraph: Forced hospitalization. Reviewed 2013)	Mental disorders or mental disability	The required treatment cannot be provided otherwise; to protect family members and third parties	–	Adult protection authority and doctors designated by the cantons	The period may not exceed 6 weeks	Yes; adult protection authority
**Sweden**	Compulsory Psychiatric Treatment Act 1991 Revised in 2000	Severe mental disorder	Need for care, unwilling/unable to decide, risk of harm to self/others due to m. disorder	Doctor in public service or Chief physician	Chief physician in 24 h	4 weeks	Yes; appeals of doctor’s decision of involuntary care are made to Administrative Court
**Finland**	Mental health act 1990	Mental disease	Need for treatment or serious danger to one’s health	Doctor employed in public health service or licensed physician or chief municipal medical officer	Within 3 days by a psychiatrist or trainee examines or three independent doctors	4 days of observation	By patient, next of kin, supervisor of commission
**Norway**	Amendment to the Mental Health Act; LOV–2012–06–22–48 Mental Health Care Act 1999 2007	Serious mental disorder	The prospects of their health being restored or significantly improved considerably reduced; it is highly probable that the condition of the person concerned will significantly deteriorate in the very near future; constituting an obvious and serious risk to their own life and health or those of others	Responsible mental health professional	Two physicians, one of whom shall be independent of the responsible institution	10 days	Yes; by the patient and their next of kin
**Denmark**	LBK nr 1729 af 02/12/2010 (Act on the use of coercion in psychiatry)	Someone who is supposed to be insane	The prospect of healing or a significant and decisive improvement in the condition will otherwise be significantly impaired or the patient presents an imminent and significant danger to themselves or others	Physician; the statement must not be issued by a doctor who is employed at the psychiatric hospital or the psychiatric ward where the hospitalization takes place	Chief physician in 48 h	–	Yes
**Romania**	Mental Health Law and protection of persons with mental disorders. Law 487/2002	Person with severe mental disorders: a person with mental disorders who is unable to understand the meaning and consequences of their behavior, so that they need immediate psychiatric help	The patient’s behavior presents an imminent danger of harm to themselves or to others; the patient does not have the psychic ability to understand the condition of the illness and the necessity of setting up medical treatment and has no legal representative or is not accompanied by a conventional representative	The family doctor or the psychiatrist specialist who takes care of this person; the family of the patient; representatives of the local public administration with attributions in the social–medical and public order domain; the gendarmerie or firemen, as well as the prosecutor; the civil court	Psychiatrist informs legal representative special committee: two psychiatrists and one specialist or a representative of civil society	Within 24 h of the psychiatric evaluation, the proposal for involuntary admission shall be examined by a special committee established within 48 h	Yes
**Russia**	Law on Mental Healthcare and Guarantees of the Citizens’ Rights in the Course of Care Provision 1993	Mental illness	Patients have to exhibit dangerous behavior toward themselves or others, they must be helpless and unable to provide for their basic daily needs, and there is a danger of “essential harm” to their mental health if they do not receive mental care	One psychiatrist	A commission of psychiatrists	48 h for assessment; documentation is sent to the local court, which has 5 days to review it; need for admission reevaluated every month for 6 months; then every 6 months; then every year	Yes; within 10 days from admission

Scotland has similar criteria but also considers the patient’s significantly impaired ability to make decisions regarding medical treatment (26). The procedures are quite different as, in Scotland, doctors can propose detention that the tribunal implements, while in England and Wales, relatives and police can also apply for third-party detention that doctors implement. The introduction of Supervised Community Treatment and Community Treatment Orders and the right to be supported by an independent Mental Health Advocate is an important recent change.

Northern Ireland (27) bases its involuntary admission on the presence of a mental disorder plus serious risk to oneself or others and the necessity of treatment (28). The first evaluator must be a psychiatrist, and proposals and validations of involuntary admission are made by doctors and appeals are made to the Mental Health Review Tribunal. In 2018, the criteria for discharge by the Mental Health Review Tribunal changed to be less restrictive.

Italy, Spain, and Sweden are the only countries in which the danger to oneself or others is not considered a criterion for involuntary treatment. However, they do require, in addition to the presence of a mental disorder, “necessity” for treatment.

An Italian law regarding “Voluntary and Obligatory Health Checks and Treatments for Mental Illness” provides that involuntary treatment can be implemented as a hospital stay: only if there are psychic alterations such as to require urgent therapeutic interventions that are not accepted by the patient, and there are no conditions and circumstances that allow alternative measures to be taken (29). This limitation of freedom takes place with a view to safeguarding another constitutional relief—that of the right to health. It is interesting to note that judgments of the Italian Court of Cassation and the Italian Constitutional Court hold different positions regarding the duty of the psychiatrist to ensure public safety (9). More specifically, there is a divide between the idea of the psychiatrist being responsible for public safety with regard to their patients and that of the responsibility being within the ambit of the police authority. Any doctor can propose a compulsory medical treatment if the conditions are met. The validation of this procedure must be done by a psychiatrist of the public service and provides for a forced 7-day, renewable, hospitalization. This document is sent to the mayor’s office, which makes a validation ordinance within 48 h.

There is no separate law exclusively for the treatment of mental disorders in Spain, but there is an insertion in a civil law that regulates the rights and dignity of the person with regard to medical and biological interventions (30). This law does not propose guidelines, nor does it indicate precise requirements necessary for involuntary treatment. Chapter II, Article 763 regards involuntary admission for mental disorders and does not speak of the involuntariness of the treatment, but of the inability to take decisions and care for oneself. As a consequence of this, involuntary admission implies that the psychiatrist responsible for the patient has the authority to order any treatment that falls under their professional responsibility (31, 32). Spain requires the first evaluator to be a psychiatrist (15). There are no defined laws regarding the maximum duration for mandatory (initial) treatments (15).

The criteria in Portugal^3^ are that the patient suffers from a serious mental disorder, which causes them to be a danger to themselves or others, refuses treatment, or is incapable of giving consent, and the lack of treatment could result in significant deterioration of their condition. The final decision of compulsory hospitalization is taken by a judge at the request of psychiatrists. The institution at which involuntary admission is carried out must communicate the admission to the court and a judge must request a psychiatric evaluation of the patient and decide within 48 h on the validity of the admission. The patient must be informed of their rights, especially with regard to appealing a decision (33).

In Greece, the regulations issued in 1992 authorized involuntary admission when there was an inability to judge one’s own health interests or if the nonadmission could lead to ineffective treatment or aggravation of the disease (34, 35). The dangerousness criterion is sufficient for an involuntary admission (15). The law provides for standard and emergency procedures. The first requires two individual assessments by psychiatrists to be completed prior to admission. Once the psychiatrists have completed their reports, the closest relative brings them to a judge authorized to issue a warrant for police to escort the patient to a hospital for admission. The emergency procedure overrides the requirement of the initial psychiatric assessments and allows a family member to apply directly to the judge. It is important to note that the emergency procedure is almost invariably the one used. In the absence of the “closest relative,” the procedure for requesting a mental health assessment is done *ex ufficio*: Upon notification by the police or concerned subject, the judge makes the request and communicates this order in writing to the police who bring the individual in for assessment. Once the individual arrives to the hospital, they are assessed by two qualified psychiatrists (35, 36).

In Belgium, the law states that protective measures may not be taken in the absence of any other appropriate treatment unless: the person concerned has a mental disorder (not including substance abuse), their condition requires urgent treatment, or they seriously endanger themselves or others. Any interested person may address a request to a Justice of the Peace who, after a hearing with the patient and all relevant persons, reviews the medical and social information, and makes a decision. During the hospitalization, the chief medical officer may prescribe an aftercare regimen for a maximum duration of 1 year (37).

In the Netherlands, the law contains two different sections for compulsory admission. The first procedure regards a brief hospitalization due to imminent danger for oneself and others and is prepared by the mayor together with a certificate written by the doctor. The other procedure is performed by a judge and relates to long-term hospitalizations for patients with severe mental disorders that constitute a danger to themselves or others, including severe negligence or social inadequacy. After discharge, treatment in the community is generally available (38).

In France, the law provides for two modalities of involuntary hospitalization: admission at the request of a third party in case of imminent peril, and admission by decision of a representative of the state. The criteria for the first are that the gravity of the mental disorder or episode makes consent impossible and the patient’s mental state requires immediate care and medical observation. The director of the medical facility takes the admission decision on the basis of two medical certificates. At the end of the initial period of admission, hospitalization can extended for 1 month, renewable. Admission by decision of the representative of the state concerns people whose mental disorders require care and compromise the security interests of the people or undermine the public order in a serious way. The representative of the state takes the admission decision in view of the psychiatric certificates (39).

In Germany, coercive interventions in psychiatry are regulated through the federal laws of guardianship, *Betreuungsrecht*, valid everywhere in the country, and in public laws with slightly different regulations in the 16 German federal states, *Bundesländer* (40). An overall tendency to emphasize civil rights is the most common characteristic of the legal mental health frameworks in Germany. The German Constitutional Court found that the law regarding involuntary pharmacological treatments was unconstitutional as written but that it could be applied in restricted circumstances to people who were unable to give consent, following a court decision based on the opinion of an independent expert (38). Compulsory admission can be required by a court order or, in some federal states, by a decision of the police, and more informally (but not infrequently) by psychological pressure from doctors and relatives. Hospitalization is defined by three types of court decisions: hospitalization in the field of forensic psychiatry, civil shelter under the guardianship law for danger to oneself or others, and civil hospitalization under public law due to acute danger for oneself or others (38). After the reunification of Germany, an improved nationwide guardianship law was passed in 1992, which shaped a new generation of state commitment laws in effect today. By adopting the basic philosophy of the national guardianship law, the Federal States adjusted their legal frameworks by placing a much stronger emphasis on the constitutional and basic human rights or safeguards of mentally ill patients as well as on the principles of community-based mental health care. The decision of the National Constitutional Court of Germany confirmed an overall “right to be ill” and exempted society at large from being responsible for improving the condition of citizens by infringing upon their personal freedom. Some State Acts permit coercive treatments in life-threatening emergencies; others restrict this only to cases in which the life of another person might be in acute danger. There are controversial positions even within Federal States and Higher Regional Court (31).

The Third Section (on the Protection of Adults) of the 2013 Swiss Civil Code states that a person suffering from a mental disorder, mental disability, or serious neglect may be committed to an appropriate institution if the required treatment or care cannot be provided otherwise, and the burden the patient places on family members and third parties and their protection must be taken into account. The Adult Protection Authority is responsible for ordering hospitalization and discharge, but the law invested the administrative authority of the Swiss Federal States (cantons) with the power to delegate the responsibility for hospitalization orders to the doctors. In 2013, a revised federal legislation came into force that was in line with international provisions. The aim was to reduce involuntary admissions and increase attention on human rights, e.g., by introducing advance directives or requiring the involvement of a legal representative. The majority of the cantons continue to delegate hospitalization powers to the doctors and the cantons assess the legitimacy of the hospitalization after 6 weeks. This study confirms that only 2% of admissions were prepared by the cantons and that the revised law did not affect the length of hospitalization (41).

The 1991 Swedish law on Psychiatric Compulsory Care established mandatory criteria for involuntary treatment: the establishment of a severe mental disorder, an absolute (essential) need for care, the patient refuses or cannot make a full judgment on the need for care, and there is a risk of harming themselves or others due to the severe mental disorder (42, 43). A decision on admission for compulsory care may not be taken without a medical certificate by a doctor in public service responsible for conducting examinations for health certificates. The question of admission must be settled within 24 h of arrival at the hospital. The admission decision is made by the chief physician of the psychiatric care unit and must not be made by the doctor who issued the health certificate. If the patient needs compulsory care beyond 4 weeks from the date of the admission decision, an application for consent must be made to the administrative court.

The Finnish Mental Health Act considers the general “right to receive care” rather than on individual civil liberties. The criteria are the presence of a mental illness, the need for treatment due to serious danger to one’s health, dangerousness, and outpatient services not being available or being inadequate (31, 44). The dangerousness criterion is sufficient for an involuntary admission (15). The patient’s opinion about their need for treatment is obtained before a decision is made, and is documented in their records. The final decision of compulsory admission requires that three independent doctors consider it justified (31).

In Norway, mental health care is provided on the basis of consent pursuant to the provisions in the Act relating to Patients’ Rights (45). On the basis of information from the medical examination, the responsible mental health professional will assess whether the following conditions for compulsory care are satisfied: voluntary mental health care has been tried, the patient has been examined by two physicians (one of whom shall be independent of the responsible institution), the patient is suffering from a serious mental disorder and application of compulsory care is necessary, it is probable that their condition will significantly deteriorate in the very near future, or they constitute an obvious and serious risk to themselves or others on account of their mental disorder. The responsible mental health professional will make a decision on the basis of an examination. The patient may appeal a decision to apply compulsory mental health care for up to 3 months after the care has terminated. Compulsory observation may not be carried out for more than 10 days from the start of the observation; then, the patient’s consent is needed. Compulsory care may also be provided on an outpatient basis when this is a better alternative for the patient.

In Denmark, the law recommends avoiding the use of coercion as far as possible (46). Admissions to psychiatric wards and pharmacological treatments should take place with the patient’s consent, and lesser interventions should be used when possible. Forced hospitalization may only take place if the patient is insane, and there is the possibility that the nonintervention would significantly compromise the healing process, or if the patient presents a significant danger to themselves or others. Anyone can call the police, who then alert a doctor to visit the patient. Involuntary admission of a person admitted to a psychiatric ward must be done only if the chief physician considers the conditions met. The chief’s decision must be taken no later than 48 h from the time of admission.

Historical sociopolitical conditions strongly influenced psychiatry and the management of the conditions of involuntarily admitted patients in Eastern Europe. In the 1990s, Eastern European countries used involuntary admissions as a political tool and a means of detention by the secret services. For example, in Romania, in the time of Ceausescu, one of the methods of oppression of political dissidents was mandatory hospitalization with politically motivated false diagnoses, made by abuses of power, which caused physical and psychic damage. Romania’s legislative decree 313/1980 established that a single psychiatric opinion was sufficient to order an involuntary hospitalization, although it is important to note that often the doctors were under government pressure (47). Article 14 of the relevant mental health law specifies that in the assessment of mental health, the psychiatrist must not take into account nonclinical criteria, such as political, economic, social, racial, and religious conflicts; family or professional conflicts; or nonconformism toward dominant moral, social, cultural, and religious mores in society (48).

In Russia, reforms to the mental healthcare system took place during the last decade of the 20th century against a background of great social and economic change. The legal regulation of mental healthcare and psychiatric care in the Russian Federation is principally through the Law on Mental Healthcare and Guarantees of the Citizens’ Rights in the Course of Care Provision. That law was developed in accordance with principles recommended by the United Nations, and came into force on 1 January 1993. The criteria for involuntary hospitalization are as follows: patients must exhibit dangerous behavior toward themselves or others, they must be helpless and unable to provide for their basic daily needs, and there is a danger of “essential harm” to their mental health if they do not receive mental care. A psychiatrist, who must provide a detailed description of the patient’s mental condition, makes the decision. A commission of psychiatrists must assess, within 48 h, whether the decision was justified, and the patient has the right to invite any specialist to participate in this process. If the admission is considered justified, documentation is sent to the local court within the next 24 h and the court has 5 days to review it. In the next 10 days, the patient, his representative, the director of the mental health facility, or an organization authorized to protect the patient’s rights may appeal against the judge’s decision regarding the hospitalization. The patient’s need for hospitalization should be reevaluated every month for the first 6 months. From then on, it should be reevaluated every 6 months. After 6 months, the commission sends the decision (regarding the necessity for continued hospitalization) to the local court, and any further continuation of treatment is approved annually by the judge (49).

### The Americas

#### USA

It has been noted in many states, particularly in California, that requiring more restrictive criteria for the possibility of involuntary hospitalization has significantly increased the number of people detained in prison (see **Table 2**). Some authors argue that favoring a dangerousness criterion, since the 1970s in the USA, has led to a criminalization of the disease (50). In the western world, detained populations have 2 to 4 times more psychosis and depression and 10 times more antisocial disorders than the regular populations. In the USA, in the 1990s, about half of the inmates had mental disorders (51). People with mental illness are detained in prison in the USA more than in any other country, and prison becomes, for them, a kind of de facto “Mental Health Asylum” (50). Contemporaneously with the promulgation of the 1963 Community Mental Health Centers Act, the USA moved away from the necessity of treatment model to the dangerousness model.

**Table 2:** Legislation, criteria, and procedures in the Americas.

Country	Legislation	Diagnosis	Other criteria	Proposal	Validation	Duration	Ability to appeal
**California, USA**	Civil Commitment laws, also known as Court–ordered–treatment	Severe mental disorder	Dangerous to self/others; unable to provide for basic personal needs for food, clothing or shelter	Members of a crisis team; other professional figures	Physicians	72 h for evaluation; 14 days of detention renewable for a maximum of 90 days	Yes
**Washington, USA**	Involuntary Treatment Act (Revised Code of Washington, Title 71, Chapter 71.05: Mental Illness)	Mental disorder or substance use disorder	Danger to self/others; danger of physical harm from failure to provide for essential human needs; severe deterioration in routine functioning as seen in loss of cognitive or volitional control	Third party	Designated mental health professional or a Court hearing	Initial detention up to 72 h; detention up to 14 days renewable for 90 or 180 days	Yes
**Alberta, Canada**	Mental Health Act: legislation for the province of Alberta 2012	Mental disorder as a disorder of thought, mood, perception, orientation, or memory that grossly impairs capacity to recognize reality or meet ordinary demands of life	Harm to oneself or others; substantial mental or physical deterioration	Anyone	Physicians; renewals require two physicians (at least one psychiatrist)	Assessment: 24 h; hospitalization: 1 month renewable for a maximum of 6 months	Yes; to the Review Panel
**British Columbia, Canada**	Mental health Act 1996	Person with a mental disorder	The mental disorder seriously impairs the ability to react appropriately to the environment; requires care in a facility to protect the person or others. Danger not required	Anyone	Physicians; police (in emergency)	First certificate: 48 h; second certificate: 1 month renewable for a maximum of 3 months	Yes; to the Mental Health Review Board. Possibility of conditional leave
**Manitoba, Canada**	The Mental Health Act 1998	Mental disease as a substantial disorder of thinking, mood, perception, orientation, or memory that grossly impairs judgment and behavior, ability to recognize reality	Risk to cause serious harm to oneself or others; the patient is unwilling or is not mentally competent to consent to a voluntary psychiatric assessment	Physicians; anyone	Medical Director; Officer of the Peace	Involuntary admission certificate 21 days. First renewal 3 months and all subsequent renewals 3 months	Yes; to the Mental Health Review Board
**New Brunswick, Canada**	Mental Health Act 2010	Mental disorder as a disorder that grossly impairs behavior and judgment and the ability to recognize reality	The recent behavior presents a substantial risk of imminent physical or psychological harm to oneself or others	Psychiatrist to the chairman of the tribunal for admission order	Assessment: physician. Admission order of the tribunal shall in writing order that the person be admitted to a psychiatric facility as an involuntary patient	Assessment: 72 h. First certificate: detention 1 month; second certificate: detention 2 months renewable for a maximum of 3 months	Yes; to the Chairman of the Review Board
**Newfoundland and Labrador, Canada**	Mental Health Care and Treatment Act, SNL 2006	Mental disorder as a disorder of thought, mood, perception, orientation, or memory that impairs judgment or behavior, ability to recognize reality	Risk to cause harm to oneself or others; possible deterioration; the patient is unable to make decisions regarding their need for treatment or care and supervision	Anyone	Judge under the authority of two certificates. Renewals made by physicians	Assessment: 72 h; first detention: 30 days, renewable for a maximum of 90 days	Yes; to the Mental Health Care and Treatment Review Board
**Nova Scotia, Canada**	Involuntary Psychiatric Treatment Act 2005, amended in 2008	Mental disorder as a disorder of thought, mood, perception, orientation, or memory that impairs judgment or behavior, ability to recognize reality	Risk to cause serious harm to oneself or others; possible deterioration; necessity of psychiatric inpatient treatment; no capacity to make admission and treatment decisions	Anyone can make a request to a Judge	Two certificates from any officer of the peace are sufficient for detention; admission by psychiatrist	Assessment: 24 h; admission: 1 month renewable for a maximum of 3 months	Yes; to the Review Board
**Ontario, Canada**	Mental Health Act R.S.O. 1990 (Consolidation Period: 2010)	Mental disorder as any disease or disability of the mind	Threat or attempt to cause bodily harm to oneself or others; lack of competence to care for oneself	Anyone (can bring evidence) to a justice of peace	Physician; Judge of peace, police	Assessment: 72 h; admission 2 weeks; renewable for a maximum of 3 months	Yes; to the Board
**Prince Edward Island, Canada**	Mental Health Act 2010	Mental disorder as a disorder of thought, mood, perception, orientation, or memory that impairs judgment or behavior, ability to recognize reality	The disorder requires hospitalization for safety of the person or others; the patient is unable to consent to psychiatric assessment	Anyone can make an application to a Judge	Judge; Officer of the Peace	Assessment: 24 h; admission: 28 days, renewable for a maximum of 12 months	Yes; to the Review Board
**Quebec, Canada**	An Act respecting the Protection of persons whose mental state presents a danger to themselves or to others 2002	Mental state that presents an immediate danger to oneself or others	Danger to themselves or others	A member of a crisis intervention unit, person having parental authority	Assessment. Practicing physician operating a local community service center; Officer of the Peace. Admission: Court	Assessment: 72 h; admission: 21 days	Yes; to the Administrative Tribunal of Quebec
**Saskatchewan, Canada**	The Mental Health Services Act 2006	Mental disorder as a disorder of thought, perception, feelings, or behavior that seriously impairs a person’s judgment, ability to recognize reality	Person unable to make informed decisions about their need for treatment and care; risk to harm oneself or others; possible physical or mental deterioration	Anyone	Assessment: Judge of the Provincial Court warrant, Constable, Peace Officer; Admission on medical certificates	First admission: 21 days renewable; long–term detention: 1 year	Yes; to the Majesty’s Court of Queen’s Bench
**Yukon, Canada**	Mental Health 2002	Mental disorder as a substantial disorder of thought, mood, orientation, or memory that grossly impairs judgment, behavior, ability to recognize reality	Threat or attempt to cause bodily harm to oneself or others; lack of competence to care for oneself, possible physical impairment	Anyone can request the Judge to make an order for involuntary examination by a physician	Validation: Judge; Officer of the Peace. Admission: physicians	Assessment: 24 h; admission: 21 days renewable	Yes; to the Supreme Court
**Jamaica**	Mental Health Care Act 2009 Parliament of Jamaica	–	Need for treatment, danger to society	Treating physician or relative of the patient (only in cases of emergency)	Director of a psychiatric hospital or psychiatric ward	7 days, renewable	–
**Argentina**	Law nr 26/657 of 2010	–	Need for treatment and danger to oneself and others	A member of the hospital team where hospitalization takes place and a psychiatrist within 48 h	Judge	30 days, renewable. After the first 7 days, a judge sends the documentation to the Body responsible for the protection of psychiatric patients	–
**Brazil **	1934	–	Inability to care for oneself, danger and moral risk to society	–	–	Unspecified duration, there are many hospitals and community residential care clinics	–

In 1964, Washington State set the conditions for involuntary hospitalization as follows. The person, other than having a diagnosed mental illness, has to pose a threat to themselves or others (actually, or as an imminent probable risk), or has an illness that impedes them from being able to fulfill their basic survival needs. In 1966, the American Supreme Court underlined the necessity for the dangerousness criterion, establishing less restrictive criteria for nondangerous patients. The dangerousness criterion is applied in a wide spectrum ranging from exclusively physical damage to a broader risk that may also include the risk of acts that are materially dangerous for oneself and others or to the ability to provide for oneself, and the risk to one’s health (1). This overlap is implicit in the “dangerousness” criterion present in much legislation, which is sometimes ascribed or not to a mental disorder. In the legislation of many countries such as Canada, the USA, and Australia, it is specified that the mental illness must be such as to seriously compromise one’s ability to react appropriately to one’s environment or to determine a deteriorated mental function (15). These elements imply a temporary loss of decision-making capacity, which represents the fundamental requirement in order to disregard the requirement of informed consent.

The conflict between the principles of beneficence and autonomy is easily overcome only when there is a clear lack of autonomy in the patient’s decisions, which *inter alia* involuntary treatment proposes to restore. In fact, the refusal of treatment can be interpreted as a symptom of the disease and the patient is supposed to hypothetically or ideally give consent. Autonomy is often assessed in favor of compulsory treatment because of the desire to restore autonomy and decision–making capacity, even though it represents a serious risk for the patient–doctor relationship and the therapeutic alliance (52).

In 1860, the legal requirement for hospitalization was the presence of a mental disorder and the prescription of a treatment. Therefore, carrying out admissions according to the principle of *parens patriae* was simple. The reaction to this led, in the 20th century, to precede the admission by request of a lawyer, and it was then reestablished that the decision had to be first taken by doctors and then approved by judges. In 1951, the National Institute of Mental Health published the “Draft Act Governing Hospitalization of the Mentally Ill,” which reestablished the psychiatrist’s power of decision at admission. In addition to redefining criteria, it implemented more guarantees for hospitalized patients and limited the duration for admissions from 2 days to 2 weeks. Extended hospitalization required a hearing before a judge, in which the patient would be assisted by a legal representative (1). Over the past decade, many national laws have been created to protect the rights of patients with psychiatric disorders or to facilitate access to care. An example of this is the Wellstone–Domenici Mental Health Parity and Addiction Equity Act of 2008, which supports the importance of a coverage for mental pathology equal to that for surgical or internal medical pathologies and which thus guarantees full access to psychiatric care, both outpatient and hospitalization. Another example is the 2012 Protection and Advocacy for Individual Rights Act, which promotes and protects the human and legal rights of people with disabilities in every US state. The establishment of procedures for involuntary hospitalization is delegated to individual state legislation.

In Illinois, anyone can petition for third–party involuntary treatment, but it must be widely documented by a first certificate compiled by a doctor or a psychologist and a second psychiatrist must examine the patient within the next 24 h and the documents must be sent to the institution’s admissions lawyers. Hospitalization may also occur by court order supplemented by a petition or certificate, by a court hearing, and upon examination by another psychiatrist. The law provides for a legal defense service and an authority that can investigate on its own initiative or in response to patient complaints of abuse. These guarantees have been met with some perplexity by psychiatrists because of the difficulty in performing involuntary hospitalizations and the substantial intrusion of legal bodies into the doctor–patient relationship (16).

California promulgated similar legislation for which members of a crisis team, or other professional figures designated by the state, could hospitalize someone in an institution designated by the state for up to 72 h for treatment and evaluation. Following that initial period, and after informing the patient of their rights, a 14-day hospitalization is permitted with medical certification, renewable for another 14 days if the patient is still a danger to themselves. If the patient is considered to be a danger to others, staff can contact the court for authorization of further treatment up to a maximum of 90 days. Each hospitalization requires a complex procedure to avoid indefinite admissions. Involuntary admissions due to severe disability require a court procedure and can last for a maximum of 1 year. The Mental Health Information Service provides patients with an ombudsman who informs them of their rights (18).

Compulsory treatment without hospitalization is a relatively modern trend in the USA. Such coercive treatment decreases the arrests of people with mental disorders, improves communication, and shortens the duration of necessary hospitalizations (1). The requirements are the presence of a mental disorder, need for treatment, a patient with poor insight and poor adherence, and a probability of danger toward oneself and others (53).

#### Canada

Most Canadian jurisdictions have evolved the dangerousness criterion into a broader “harm” criterion and introduced, as an alternative, “likelihood of significant mental or physical deterioration.” These broader harm criteria have been found by courts to be in accordance with the Canadian Charter of Rights and Freedoms (54). Three provinces also require the person not to be fully capable of making an admission or treatment decision. Often the judge represents the first interlocutor for initial requests by anyone and can provide an examination order as is frequently necessary for a treatment decision when there is difficulty in obtaining the consent of the person or their representative. Admission validation is often carried out by the judicial authority on the basis of medical certificates but is sometimes done directly by doctors (e.g., as in Nova Scotia). All provinces provide for reviews at the request of the patient or other interested persons, and appeals against the decision of the review board are almost always foreseen. The process of assessing the legality of hospitalization (having a court hearing and the right to consult a lawyer) sometimes has a beneficial effect on the patient’s acceptance of treatment. Thus, the opportunity to make extensive use of the law is an important therapeutic factor (55).

#### Central and Latin America

To make the difficult transition from psychiatric hospitals to general hospitals and local services, the Jamaican government has set up a network of specialized nurses, called “mental health officers,” who are responsible for providing follow–up assistance to patients after discharge. Involuntary treatment is used as little as possible, and hospitalization durations are generally short and limited to the time of crisis. However, popular access to treatment and medicines is limited and drugs currently in use in western countries are hard to find. The health care service keeps a list of patients who have difficulty following prescribed therapy for this lack of access, or for lack of adherence to treatment, and of all patients who missed their last checkups. This list is used to send nurses to the patient’s home to ensure their health conditions (56).

Argentina created a new law on the rights of psychiatric patients in 2010, which specifies that 10% of health expenditure must be used for a transition from custodial to community psychiatry. It also prohibits the construction of new psychiatric hospitals, instead shifting efforts to placing patients in beds in the general hospitals and strengthening services in the territory. Hospitalization is to be carried out under conditions of urgency, and the two criteria for a compulsory admission are as follows: danger for oneself or others, and a patient’s lack of understanding of their state and a consequent inability to express consent to the care (57). Contrarily, in Brazil, the criteria for involuntary treatment are expressed in a law of 1934 (but are not explicitly stated in the current law) as imminent danger to oneself or others, “moral” risk to society (for example, inadequate sexual or financial behavior), or inability to take care of oneself. Access to psychiatric treatment is not ensured in Brazil, and patients often remain at home in desolate conditions, on the street, or even sometimes in prison. There are three types of hospitalization: voluntary admission where the patient consents to treatment, involuntary admission where there is only partial consent, and forced admission in the event that the patient denies consent. Forced hospitalization occurs when necessary, while involuntary admission must be authorized by a judge (58).

### Oceania and Asia

New Zealand requires the presence of serious danger to the safety of oneself or others, seriously diminished capacity to take care of oneself, or serious danger to their health (see **Table 3**). Anyone may apply to the Director of Area Mental Health Services for an assessment, which is determined by doctors as the Compulsory Treatment Order (community treatment order or an inpatient order) is decided by a court. The Mental Health Act of 1992 introduces community treatment orders in New Zealand. Clinicians often consider them to be a useful strategy for patients with schizophrenia and major affective disorders, as many scholars have identified the need to move beyond hospital utilization rates as a measure of efficacy (59).

**Table 3:** Legislation, criteria, and procedures in Asia and Oceania.

Country	Legislation	Diagnosis	Other criteria	Proposal	Validation	Duration	Ability to appeal
**New Zealand**	Mental Health (Compulsory Assessment and Treatment) Act 1992 No 46 (as at 21 February 2018)	Mental disorder: abnormal state of mind characterized by delusions, or by disorders of mood or perceptions or volition or cognition of such a degree that fit criteria for compulsory assessment and treatment	1) Poses a serious danger to the safety of that person or of others or 2) seriously diminishes the capacity of that person to take care of himself or herself or poses a serious danger to their health	Anyone duly authorized may apply to the director of area Mental Health Services for an assessment (with a certificate by a health practitioner). The duly authorized officer or the director of Area Mental Health Services must ask an assessment examination by a psychiatrist or a medical practitioner.	Assessment health practitioner, responsible clinician. Compulsory Treatment Order: The Court (apply by the responsible clinician)	First period of assessment: 5 days. Further period of assessment: 14 days. Compulsory Treatment: 6 months. After review possible renew: 6 months twice. If the court then further extends the order, the extension shall have effect indefinitely	Yes; to the family court Judge or any District Court Judge
**Australia**	Different legislation for involuntary commitments and treatments among Australian Jurisdictions	Mental illness	Risk of serious harm to the person or to others and provision of treatment for that illness	Procedures vary among jurisdiction. For example, Victoria Mental Health Act states that an Assessment Order can be made by a registered medical or mental health practitioner to enable a person to be compulsorily examined or detained	The assessment must be made by an authorized psychiatrist who can issue a Temporary treatment Order to detain and treat the patient in a designated mental health service. Treatment order is made by the tribunal	Assessment Order: 72 h Temporary treatment Order: 28 days Treatment Order: 6 months	Yes
**Israel**	Law on care of the mental health patients 1991	Inability to exercise judgment and to criticize reality	Immediate risk to endanger themselves or another person. Refuse to be examined by a psychiatrist	District psychiatrist	–	7 days, extendable by the chief psychiatrist or District Psychiatric Committee	Yes; to the Psychiatric committee
**Taiwan**	Mental Health Act of Taiwan 1990, amended in 2007	Psychotic state	Unable to adhere to treatment; danger to themselves or others	–	Two designated psychiatrists	60 days; possible extension of another 60 days	–
**China**	Mental health law of the People’s Republic of China, 2012	Severe mental disorder	Self-harm, behavior that harmed or endangered the safety of others	Family members, employers, local police	One psychiatrist	Not specified	Yes
**Japan**	Law Related to Mental Health and Welfare of the Person with Mental Disorder, 2006	Compulsory admission: Mental disorder	Risk to harm themselves or others	Any party or the police has to notify the person to the prefectural governor	Two designated physicians	Initially 4 weeks; continuation not defined	Yes; to the Psychiatric review committee
		Admission for medical care and protection: Mental disorder	–	The person responsible for their protection (guardian)	One designated physician		
**India**	The Mental Healthcare Act, 2017 NO. 10 OF 2017	Mental disorder	Recently threatened or attempted to harm themselves, behaved in a violent manner toward others, have caused someone to fear for their safety, to have shown an inability to take care of themselves at a level which put them at risk of harm	Patient’s representative	Mental health professional or a general practitioner for the initial admission; after that assessment by two separate psychiatrists	30 days for the initial admission; after that 90 days, renewable up to 180 days	Yes; to the Concerned Board
**Korea**	Mental health Act No. 12935, 2014	Severe mental illness	Danger to harm themselves or others	Anyone with the consent of a physician and a police officer	One psychiatrist	Emergency hospitalization: 72 h; after psychiatric assessment: up to 6 months	Yes
**Malaysia**	Laws of Malaysia, ACT 615, Mental Health act 2001	Mental disorder	Admission necessary for assessment and treatment to protect health and safety of patient and others	Request from a relative, recommendation of a medical officer or a registered doctor	Medical director of the psychiatric hospital with the assessment made by medical officer or registered practitioner	24 h for the initial assessment; after that 1 month extensible up to 3 months	Yes; the patient or relatives can submit a request to the medical director for the discharge
**Iran**	No separate mental health legislation	Severe mental disorder	Serious risk to themselves or others	–	Forensic Doctor	Up to 2 months	–
**Pakistan**	Mental Health Ordinance for Pakistan 2001	Mental illness	Admission necessary for the safety and health of the patient or the protection of others	–	Two medical officers, one of whom shall be a psychiatrist	Admission for: 1) Assessment: 28 days; 2) Treatment: 6 months 3) Emergency admission: 72 h 4) Emergency holding: 24 h	Yes; before the Court
**Thailand**	Mental Health Act, B.E. 2551 (2008)	Mental disorder	Threatening condition and need for treatment	Family member, caregivers. Anyone who thinks that a person needs treatment should report to an administrator or police officer who must bring the patient to the nearest hospital	A doctor and a nurse for preliminary diagnosis; infirmary board of the hospital for the admission	48 h for the evaluation; initial duration 90 days, extensible for another 90 days	Yes; to the Appeal commission

In Australia, each jurisdiction has its own mental health act that regulates the involuntary commitment and treatment of people suffering from mental illness. Although every jurisdiction has its own definitions, generally the presence of a mental illness, a risk of serious harm to the person or to others, and the provision of treatment for that illness are required. Hospitalization has to be the least restrictive alternative to ensure appropriate treatment to the patient (60). Procedures for compulsory assessment and admission vary as well among jurisdiction: for example, the Victoria Mental Health Act No. 26 of 2014 states that an Assessment Order can be made by a registered medical or mental health practitioner to enable a person to be compulsorily examined or detained after an evaluation by an authorized psychiatrist. A Temporary Treatment Order is made by an authorized psychiatrist, and a Treatment Order is made by a tribunal. A patient may seek a second psychiatric opinion at any time.

The new Statue of the State of Israel allows for involuntary treatment for people with mental disorders that cause deterioration in judgment or in the ability to recognize reality, which causes severe emotional injury to others. The district psychiatrist can order an involuntary psychiatric evaluation of the subject. Hospitalization shall last no more than 7 days, with the exception of the possibility that the chief psychiatrist of the district may extend the admission for another week. An appeal against the district psychiatrist’s decision can be made by any person (not necessarily by the patient or a relative) to the Psychiatric Committee (61).

In Taiwan, the Mental Health Act, introduced in 1990 and amended in 2007, legally defined the criteria for involuntary treatment as a patient in a psychotic state who is unable to adhere to treatments and is a danger to themselves or others (62). Compulsory hospitalization for severe mental illness should be determined by two designated psychiatrists (63). Psychiatrists in Taiwan expect family members to participate in treatment decisions: Patients are often persuaded by family members to sign for admission and treatment, so the proportion of involuntary hospitalizations (7.3 per 100,000) is low, compared with other developed countries (62).

In China, when persons with a suspected mental disorder harm themselves or others, or endanger the safety of others, close family members, employers, or local police shall take them to a medical facility for a psychiatric assessment (64). A psychiatrist must make the admission decision. There are no separate legal definitions for hospitalization and involuntary treatment, and mandatory treatment beyond hospitalization is not allowed. Treatment can be imposed if the guardian or family approves; otherwise, it should not be administered. If the patients or their guardians disagree with the result of the diagnostic assessment, they may request a diagnostic reassessment and an independent, legally binding certification of the case (64). The law does not specify the initial interval or the revaluation interval (15).

In Japan, there are two types of involuntary hospitalization: a compulsory admission indicated by two designated doctors, and admission for medical care and protection (65). Only the first of these requires the patient to be a danger to themselves or others if they are not hospitalized. In the first type of compulsory admission, the patient must have a mental disorder and be at risk to hurt themselves or others unless hospitalized (66). The second type of involuntary admission states that the administrator of the mental hospital may admit a person without their consent if the person responsible for their protection consents to such hospitalization, based on the examination by the designated physician. The initial duration of involuntary treatment is 4 weeks, the duration of continuation is not defined, and, outside of hospitalization, there is no allowance of other mandatory treatment. Decisions are reviewed by a psychiatric review committee whose members take into consideration any discharge requests from the patient or their guardians (15).

In India, according to the Mental Healthcare Act nr. 10 of 2017, the person must suffer from a mental disorder of such severity to put themselves or others at risk of harm and have shown an inability to take care of themselves (67). A mental health care professional may admit a patient to an institution, upon request by the patient’s representative, if the criteria are met in a recent assessment, by a mental health professional, or by a general practitioner. The involuntarily admitted patient may request a review of the admission decision by the “Concerned Board.”

The Korean Mental Health Act No. 12935 of 30 December 2014 allows for involuntary admission if a psychiatrist determines that the patient suffers from a severe mental illness of such a grade and nature that it requires hospitalization in a mental health institute and if the hospitalization is necessary for the health or safety of the patient or others. When the situation is particularly urgent, anyone who suspects that a mentally ill person presents a risk to themselves or others may request their emergency hospitalization with the consent of a physician and a police officer. The duration of emergency hospitalization can last up to 72 h, before requiring a psychiatrist to establish the need for continued admission (68).

In Malaysia, the criterion is that a patient must have a mental disorder that is grave enough to require admission to psychiatric hospital for assessment or treatment in the interest of their health or safety or to protect others. The law specifically states that no consent is required to administer psychiatric drugs. The patient or relatives can submit a request to the medical director for the discharge of an involuntary patient. A patient who has been discharged may be required by the medical director to undergo community care treatment at a government community mental health center (69).

The situation in Indonesia for persons with mental illness is far from satisfying from a human rights point of view. Basic mental health services are unavailable in many parts of the country, and the primary psychiatric treatments are custodial in nature. Involuntary treatment is common even though there is no real legal basis for it. A person can be brought to a hospital and committed without their consent by anyone who feels negatively affected by their behavior (70).

The lack of human resources and governmental investment in mental health services in Cambodia forces the families of mentally ill patients to deal with their illnesses without the support of adequate medical assistance. When mental disability is severe, many family members are forced to resort to chaining or caging patients as a solution (a phenomenon that seems to involve 10–40% of psychiatric patients). Many mentally ill patients are put in prison or in detention facilities that operate outside the criminal system and where drug addicts and other “undesirables” such as homeless people, prostitutes, and the mentally disabled are illegally detained. Individuals who are detained are not accused of any crime and do not have the right to confer with a lawyer or to request a review of their detention. Treatment in these centers is brutal: involving chains, beatings, and overcrowding, and people can be detained for months or years (71).

Iran requires the presence of a severe mental disorder and a serious risk to themselves or others. A forensic doctor must carry out the assessment regarding whether the criteria for involuntary treatment are present, and if so, they shall determine the duration of the admission, up to a maximum of 2 months (72).

The “Mental Health Ordinance of 2001” regulates mental health care in Pakistan, and the duration of involuntary treatment depends on the context. A patient can appeal against an admission decision before the Court. This new law reduced the period of forced detention by the police and magistrates from 10 h to a maximum of 72 h, which has minimized abuse of the system. Another important clause provides for a psychiatric assessment for all blasphemy defendants to ensure protection and to curb the high number of psychiatric patients who are punished and tried under the “blasphemy laws” (73).

A patient in Thailand can be subject to mandatory treatments when they suffer from a mental disorder and are in a threatening condition and need treatment. If the patient is unable to consent to treatment, consent is provided by a family member or caregiver. If the presence of a mental disorder is ascertained, the “Infirmary Board” decides whether to admit the patient or order them to seek treatment outside if they are not in a state of threat. The patient or caretaker can apply to the appeal commission within 30 days of admission (74).

### Africa

The current mental health situations on the African continent vary widely. We roughly distinguish sub-Saharan Africa from the countries of the Maghreb. The latter, together with Somalia and Sudan, have joined the WHO Mental Health Atlas, and some of them (Algeria, Egypt, Morocco, and Sudan) have an autonomous legislative system on mental health. These countries have committed themselves to reviewing the principles on which mental health laws are based in order to adapt to WHO standards (14). Despite these intentions, the rights of the sick are not often recognized and respected in many countries. Often, the main criterion for which a patient is hospitalized is the state of danger to themselves or others, although requests by family members who cannot take care of the patient at the time of crisis are another frequent reason for hospitalization (75).

In Algeria, the first mental health law dates back to French colonization. Before that, the sick were brought to the *maristans*, asylums dating back to the Ottoman Empire. At the beginning of the colonial era, patients were instead brought to psychiatric hospitals in the south of France until 1912, when psychiatric hospitals were built in Algeria. After independence, Algeria presented a bill that essentially followed the French model. In 1985, a second mental health bill came into force, which still applies today. According to this law, there are two types of involuntary admission: hospitalization by written request of a family member or legal guardian, and admission required by the local governor (*wali*), based on a medical certificate attesting an imminent risk to the patient or others or that the patient is currently unable to give his or her consent. The involuntary hospitalization has a maximum duration of 6 months and can be renewed by a doctor who must submit the request for continuation of treatment to a commission headed by the *wali* (76).

In Libya, the health system is inadequate and without resources due to the current civil war. There are only two psychiatric hospitals in the country, and the conditions of hospitalized patients are very poor: hygiene is neglected, procedures are antiquated and implemented without taking into account the rights of patients, and hospitalizations (almost all involuntary) are transformed into long-term stays of months and sometimes years. Patients often come to services with chronic illnesses and often late, only when their family is no longer able to manage them. This is due mainly to the stigma associated with mental illness. Family members initially prefer to consult healers and then turn to the family doctor, and only when these attempts fail, they turn to a psychiatrist. Libya’s current legislation on mental health came into force in 1975 and has not been revised (77).

The situation is also precarious in sub–Saharan Africa, although many countries try to adapt to the principles and criteria of the South African Mental Care Act of 2002. Unfortunately, the scarcity of resources invested in individual national health systems and the deeply rooted traditional cultures have not allowed many countries to protect and regulate the rights of psychiatric patients yet. It should be noted that some countries, such as South Africa, are moving toward modernization. In many others, hospitals and health care are present almost exclusively in large cities, leaving rural areas almost completely lacking in services (78). The belief present in many rural villages is that mental illness is the work of a djinn (a spirit) that possesses the person and upsets the mind. This causes people to turn to ancient traditions and rituals in the hope of expelling the malignant entity. In many countries, for example, the sick are stripped and chained to poles outside the houses; in others, healers are called to try to free the sick through rituals of black magic (79).

There is an interesting story of Gregoire Ahongbonon called “the African Basaglia,” a tire maker born in Benin who, during a trip, noticed a malnourished boy chained because of his illness. Ahongbonon released him and, with the help of a nurse, began to free many others and to form a kind of community care for psychiatric patients. Currently, there seem to be thousands of people living, or who have lived, in such communities. However, the comparison with Basaglia seems in reality not very fitting. Ahongbonon did not propose a passage from a custodial to a community psychiatry but instead acted by linking popular beliefs and legends to a possibility of medical care; in this way, perhaps, we could think him more similar to Pinel (80).

### Criteria for Involuntary Care With Regard to Risk of Suicide

It is convenient to emphasize that in the jurisprudence inherent to the treatment of mental illness, the danger for oneself and others is generally considered nearly equal. The psychiatrist is responsible for the prevention of self-injurious acts as well as those who injure others. This naturally raises the problem of individual freedom even in the absence of material damage to third parties. Suicide is not a crime in the vast majority of legal systems, and India has introduced a new bill, which mentions the decriminalization of suicide attempts (81). It states that there is a serious stress in those who make the attempt, and such people should not be tried and punished. Furthermore, it is noted that the government should provide treatment and rehabilitation for these persons, and take measures to reduce their suicidal risk (82). In Nepal, attempted suicide is illegal: People who attempt suicide are imprisoned or fined (83). In the Western world, current legislation regarding suicide has become less punitive (84).

The extreme measures of prevention demanded of psychiatrists are a consequence of the times following Esquirol (1821) who interpreted suicide as a medical problem that occurs as the consequence of a mental disorder. There is an open debate on this univocal interpretation of suicide, although this interpretation is supported by many Western academics. Moscicki states that a psychiatric disorder is a necessary condition for suicide, and Jamison asserts that there is “the unequivocal presence of a serious psychopathology in those who die by their own hand.” Contrarily, some authors, especially in Asia, criticize the medicalization of suicide by claiming that only some of the people who commit suicide suffer from mental disorders. A report in Korea states that “the current suicide epidemic in Korea has social origins” (84). In the WHO Report on Preventing Suicide: a Global Imperative, involuntary treatment does not figure in the prevention of suicide, although it is allowed in many countries, including England and Wales. Compulsory medical treatment can sometimes actually increase suicide risk by discouraging treatment requests for fear of being detained. Regardless, the WHO recommends that requests for help should be encouraged. Among other things, the high risk of suicide was noted after resignation due to the experience of discrimination and dehumanization of hospitalization (85).

Article 14 of the UN Convention on the Rights of Persons with Disabilities (CRPD), entitled: Liberty and security of person, provides that:

“1. States Parties shall ensure that persons with disabilities, on an equal basis with others:

a) Enjoy the right to liberty and security of person;

b) Are not deprived of their liberty unlawfully or arbitrarily, and that any deprivation of liberty is in conformity with the law, and that the existence of a disability shall in no case justify a deprivation of liberty.

2. States Parties shall ensure that if persons with disabilities are deprived of their liberty through any process, they are, on an equal basis with others, entitled to guarantees in accordance with international human rights law and shall be treated in compliance with the objectives and principles of the present Convention, including by provision of reasonable accommodation” (86).

According to that article, the criterion of danger to oneself and others, linked to actual or perceived impairment, is not a sufficient reason for compulsory treatment. However, most legal systems (excluding, for example, Italy and Spain) accept that prohibiting compulsory treatment contravenes people’s rights, such as their right to treatment to avoid suicide. In fact, this prevention constitutes a frequent reason for obligatory treatment. In a study conducted in Belgium on 346 patients subject to involuntary treatment, 45.1% of them were considered to be a danger to themselves (87).

There are two clinical problems in suicide prevention, one of which pertains to the difficulty in predicting suicide risk. A recent meta-analysis revealed that in a 5-year follow-up, nearly half of suicides were considered low-risk patients, while 95% of high-risk patients did not die by suicide (85). The other problem considers that preventing suicide with coercive measures can hinder a psychotherapeutic path. This is due to the repercussions of a communicative and emotional gap that the coercive measure contains, which is often not easy to elaborate in the relationship. In this context, the symbolic and relational, rather than legal, significance of the Ulysses contract is important as it provides for a prior consent to treatment (10). Psychiatrists can sometimes find themselves in a painful contrast between their legal responsibility and the desire to cure.

The Vienna Declaration and Program of Action on Human Rights, adopted by the World Conference on Human Rights in June 1993, provided at paragraph 64 of Chapter 6 (“The Right of the Disabled Person”) that “the place of disabled persons is everywhere.” Following that principle, the CRPD, adopted on 13 December 2006, and its later interpretation through the Committee’s Guidelines on Article 14 of the Convention (adopted September 2015), continue the long road to full equality, respecting the particularities of disabled people (see, in particular, paragraph e of the Preamble and Articles 3, 5, 12, 14, and 15 of the CRPD). These rules are intended to combat society’s fear of disability and mental illness, and to eliminate the consequent stigma. National legislators, psychiatrists, and jurists are called to follow this road. With regard to the broad debate, generated by the extreme positions taken by the CRPD Committee (e.g., that involuntary admission and involuntary treatment are illegal) in light of the above considerations, we believe jurists and psychiatrists have to interpret the national provisions, as far as possible, in line with the CRPD and its Guidelines (88–91). Any justified exception must be a last possible course of action. On the other hand, we should probably consider as a positive exception also those situations in which involuntary admission and treatment concern people in a state of social marginalization or existential loneliness for which this option may paradoxically represent the only possibility of access to the treatments that potentially open to a path without which the situation could become increasingly painful and dangerous.

## Discussion

Although most countries around the world are trying to make progress in psychiatric procedures and legislation, we have seen how varied the situation is. In Africa, the lack of progress can be attributed to multiple causes. For example, stigma is still strongly present in many countries (as it is even in Western countries); famines, epidemics, wars, and political instability often do not allow for focus on improvements; and lack of funds as well as of the proper mentality and infrastructure also contributes to the stagnation.

In Latin America, the situation is different: Many governments are concentrating their efforts on transforming structures, making them more livable, and setting up departments within general hospitals for psychiatric patients. Some countries have allocated a considerable share of health funds to psychiatry. Despite this, we are still far from the passage in the mind and in the material reality from a custodial psychiatry to a psychiatry that protects the rights of the patient while providing appropriate care.

In Europe, legislation emphasizes the exceptional nature of compulsory medical treatment. Australia and some Asian countries comply with western regulations, while in other Eastern countries, the mentality and regulatory framework remains in the custodial mold. It is, however, true that the treatment of the mentally ill is a fundamental element for the assessment of the pluralist democracy level of each legal system. In less developed countries, the stigma of the psychiatric patient determines that the treatment is, in fact, entrusted completely to the family and contact with the psychiatrist takes place only in situations of imperative urgency, which often means that the first contact of care results in a coercive measure. In these areas, greater social support for patients with mental illness would be desirable. As has been implemented already in some countries, it is important, globally, to increase from early on society’s awareness of mental illness so that people with mental disorders have a lived experience of greater reception and can cope (as far as possible) with mental pain in a more supportive group manner. There are two critical issues concerning mandatory treatment: the breach of the self-determination principle and the risk of breakdown of the therapeutic relationship. If the context of mental illness is the only area in which the refusal of treatment is often identified as a symptom of the disease, it is important to evaluate, in a multidimensional view, the decision-making capacity of the patient (9).

The injury to self-determination causes a wound that is difficult to process. In this regard, it is noteworthy that even patients who subsequently considered a compulsory admission to be justified maintained a feeling of anger about the event. In this light, advance treatment directives (such as Ulysses contracts) can play a significant role: In fact, various authors highlight how they can help promote self-determination and the ability of the patient to make decisions (92). The ability to imagine a potential future crisis and its resolution can enhance the patient’s insight on their pathology. It seems that advanced directives reduce involuntary treatments, helping patients better understand the need for crisis prevention and ways to decrease violent acts. This early consent is a valid tool for deescalating crisis: Knowing that a given intervention, even if it causes discomfort, has previously been agreed to by the patient in discussion with medical staff makes it more acceptable compared to an *ex novo* coercive intervention.

The central role of communication in therapeutic relationships and its substantial destruction in emergency coercive interventions lead one to consider the resumption of the interrupted communication postcrisis or the possibility of using advanced directives. The greatest risk of coercive intervention in the psychiatric field is constituted by the absence of the recognition of emotions during the implementation and by the consequent communication impasse between the psychiatrist and the patient. This could create a lacuna in the experience of both the patient, whose crisis is emptied of meaning while requiring urgent intervention, and the psychiatrist, which can be difficult to process for both of them.

It would be interesting to explore the therapeutic role of procedures in which the patient communicates with various interlocutors. The more articulated procedure actually determines a dialogue with a significant relational meaning and is opposite to the abandonment anxiety that involuntary admission can cause. Hospitalization, in addition to representing a physical distance from the family, often represents an interruption of communication for the patient. This interruption, inherent in involuntary admission, can also stir up the persecutory anxieties of being a monster excluded from human assembly. Procedures that provide for discussions regarding hospitalization can acquire the symbolic value of a reintegration into the patient’s humanity.

### Patient Experiences

Some studies underline the patient’s experience of involuntary treatment, in particular with regard to the respect for their dignity. The Universal Declaration of Human Rights gives dignity a central role, stating “all human beings are born free and equal in dignity and rights.” In the UK, Mental Health Act of 1959^4^ emphasizes, in addition to the right of the person to receive medical treatment and the need for public protection, the right to dignity and freedom (93). This act was accepted as an act of social welfare. Title I of the Charter of Fundamental Rights of the European Union^5^, entitled “Dignity,” is composed of five articles. Article 3, entitled “Right to the integrity of the person,” states in paragraph 2(a) that “[I]n the fields of medicine and biology, the following must be respected in particular: (a) the free and informed consent of the person concerned, according to the procedures laid down by law” (94). Article 4 of the ECHR, identical to Article 3 of the CEDU, prohibits degrading or inhuman treatment. The sense of loss of power and autonomy, the reduction of self–esteem, and the main desire to be treated with dignity and respect by the staff can be seen in the semistructured interviews of patients who have been subject to involuntary treatments. Reduction of the coercive element and patient involvement contributes to reduction in the feeling of loss of dignity (93). Therefore, advance directives, which represent an important relational element, lessen feelings of coercion (95). The painful consideration of the relativity of the concept of freedom is opportune in cases in which, paradoxically, dignity would be more affected by the lack of treatment of marginalized people living in degrading conditions, easy victims of criminality. This also applies to the suffering of patients and third parties for violent acts carried out in the acute phase.

Hospitalization seems to be associated with high levels of perceived humiliation and consequent anger (96, 97). The lived experience of humiliation seems to be greater in schizophrenic patients with depressive comorbidity and in those with low education and/or lacking employment (98). This element has inevitable consequences on the therapeutic relationship and on the patient’s adherence to the treatment plan. Therefore, future efforts must be made to minimize the use of coercive measures that breach the principle of self–determination and impede doctor–patient communication. A Swedish study observed that hospitalization and coercive care are experienced by the patient as a loss of freedom, where the patient is not involved and in which no one cares for them or explains what is happening. Alternatively, some patients feel respected and cared for by the staff, which may facilitate their request to take personal responsibility for being involved in their care. Differently, sometimes the patient can feel relieved at not being involved in the decision–making process as it absolves them from duties and responsibilities when they are not well (43). In any case, objective legal status and subjective feelings are not equivalent: Formal legal status is not the only etiological factor in the variability of the perception of coercion (95). In general, if it can be assumed that the greater the sense of coercion, the worse the clinical outcome for the person in care, other results could be that negative hospitalization experiences do not influence the outcome (9, 99). Informal coercion also plays an important role. Psychiatrists interviewed in the study considered informal coercion to be effective in the therapeutic process and with future adherence. It should be noted that the use of informal coercion can be underestimated as it is sometimes used unintentionally. When informal coercion is accompanied by high levels of perceived coercion and a sensation of disparity, the therapeutic relationship may be affected by an interruption of the therapy. Conversely, benefits such as increased adherence, promotion of clinical stability, and avoidance of relapse occur when there is a combination of a low level of perceived coercion and a sensation of fairness and justice (100). A study conducted in Pennsylvania and Virginia observed that approximately 10% of the voluntarily admitted patients reported that they had felt forced into admission during negotiations with the hospital staff (101). Gardner and Lidz noted that even patients who considered their hospitalization justified continued to experience anger with regard to their admission as a result of the damage caused by coercive elements and the consequent loss of autonomy (102).

### Role of the Family in Health Treatments

In many parts of the world where health systems are limited or nonexistent, family members are the only resource for people with mental disorders. While in the European countries there is great attention to privacy, in other traditions this aspect takes on less importance. In many parts of India, for example, a family member is required to stay in the hospital to ensure that the patient does not leave, to cook for them, and to provide for the patient’s hygiene. This role, taken on by the family member, infringes on the patient’s right to privacy. Most laws have clauses allowing for involuntary treatment upon request by family members, although their involvement can put them in a position of conflict with the patient (103). The familial relationship is an important element even when not related to decision–making power but when the family has the ability to generate the initial request for the hospitalization. Often, the patient’s family members prefer to be kept out of the implementation of involuntary treatment because of the repercussions for their relationship. The differences among countries with regard to pressure on hospitalization are noteworthy. For example, in Europe, relatives in Bulgaria exert more pressure on admissions, while in Italy, relatives tend to avoid requesting hospitalization (104). In Japan, compulsory hospitalization is generally ordered by the Prefecture Governor, but if a family member consents, all that is required is that the patient has a mental disorder and needs hospitalization. In fact, traditionally, the family is the primary decision-making body for its members. In India, hospitalization is carried out by request of a relative or a friend if two doctors agree on the need in the interest of the sick person (15).

The singer Frank Zappa once said: “the biggest problem in the world is mental health.” To date, many countries are tackling this problem by adapting their legislative systems to offer potential prevention strategies and appropriate care for people with mental disorders. There is much to do in the psychiatric field to ensure care, dignity, and rights for patients, but perhaps, despite the economic disparities, cultural traditions, and related stigma in various countries, we begin to take the first steps in the right direction.

## Author Contributions

All authors listed have made a substantial, direct, and intellectual contribution to the work, and approved it for publication.

## Conflict of Interest Statement

The authors declare that the research was conducted in the absence of any commercial or financial relationships that could be construed as a potential conflict of interest.

## Footnotes

1 The Community Mental Health Centers Act of 1963.

2 Danish law nr. 331 of 24 May 1989 and following revisions.

3 Law 36/98.

4 Revised from 1983 to 2007.

5 With effect on 1 December 2009, binding 28 Member States (including the United Kingdom).
